# The Views and Experiences of Integrated Care System Commissioners About the Adoption and Implementation of Virtual Wards in England: Qualitative Exploration Study

**DOI:** 10.2196/56494

**Published:** 2024-11-27

**Authors:** Laura J McGowan, Fiona Graham, Jan Lecouturier, Louis Goffe, Carlos Echevarria, Michael P Kelly, Falko F Sniehotta

**Affiliations:** 1 NIHR Policy Research Unit in Behavioural and Social Sciences Population Health Sciences Institute Newcastle University Newcastle upon Tyne United Kingdom; 2 Health Determinants Research Collaboration Gateshead Council Gateshead United Kingdom; 3 Newcastle upon Tyne Hospitals NHS Foundation Trust Newcastle upon Tyne United Kingdom; 4 Department of Public Health and Primary Care University of Cambridge Cambridge United Kingdom; 5 Centre of Preventive Medicine and Digital Health Division of Public Health, Social and Preventive Medicine Heidelberg University Mannheim Germany

**Keywords:** virtual wards, remote monitoring, whole systems, qualitative, implementation science, integrated care system, England, digital technology, acute care, clinical practice, semistructured interviews, thematic analysis, patient-centered care, hospital-centric language, eHealth, health services

## Abstract

**Background:**

Virtual wards (VWs) are being introduced within the National Health Service (NHS) in England as a new way of delivering care to patients who would otherwise be hospitalized. Using digital technologies, patients can receive acute care, remote monitoring, and treatment in their homes. Integrated care system commissioners are employees involved in the planning of, agreeing to, and monitoring of services within NHS England and have an important role in the adoption and implementation of VWs in clinical practice.

**Objective:**

This study aims to develop an understanding of the acceptability and feasibility of adopting and implementing VWs in England from integrated care system commissioners’ perspectives, including the identification of barriers and facilitators to implementation.

**Methods:**

Qualitative semistructured interviews were conducted with 20 commissioners employed by NHS England (NHSE) in various geographic regions of England. Thematic analysis was conducted, structured using the framework approach, and informed by the Consolidated Framework for Implementation Research. The COREQ (Consolidated Criteria for Reporting Qualitative Research) guidelines were followed.

**Results:**

Four overarching themes were identified reflecting the acceptability and feasibility of key adoption and implementation processes: (1) assessing the need for VWs, (2) coordinating a system approach, (3) agreeing to Program Outcomes: NHSE Versus Organizational Goals, and (4) planning and adapting services. Commissioners expressed the need for system-level change in care provision within the NHS, with VWs perceived as a promising model that could reform patient-centered care. However, there was uncertainty over the financial sustainability of VWs, with questions raised as to whether they would be funded by the closure of hospital beds. There was also uncertainty over the extent to which VWs should be technology-enabled, and the specific ways technology may enhance condition-specific pathways. Differing interpretations of the NHSE instructions between different health care sectors and a lack of clarity in definitions, as well as use of hospital-centric language within national guidance, were considered hindrances to convening a system approach. Furthermore, narrow parameters of success measures in terms of goals and outcomes of VWs, unrealistic timescales for planning and delivery, lack of interoperability of technology and time-consuming procurement procedures, liability concerns, and patient suitability for technology-enabled home-based care were identified as barriers to implementation. Motivated and passionate clinical leads were considered key to successful implementation.

**Conclusions:**

VWs have the potential to reform patient-centered care in England and were considered a promising approach by commissioners in this study. However, there should be greater clarity over definitions and specifications for technology enablement and evidence provided about how technology can enhance patient care. The use of less hospital-centric language, a greater focus on patient-centered measures of success, and more time allowance to ensure the development of technology-enabled VW services that meet the needs of patients and staff could enhance adoption and implementation.

## Introduction

### Background

Health and social care services across England are currently facing increasing waiting times and unmet demand [1], creating high levels of system pressures that negatively impact both patients and health care staff. What have been defined as avoidable hospital admissions along with prolonged in-patient stays cause patients distress, limit hospital bed capacity, and are costly to the National Health Service (NHS; the publicly funded health care system in the United Kingdom). Older people and those from socioeconomically disadvantaged areas are at a greater risk of emergency admissions [2,3]. The emergence of COVID-19 has brought new urgency to the adoption of strategies that increase hospital bed capacity and reduce the risk of hospital infections.

Virtual wards (VWs) are models of care delivery whereby patients receive the care they need at home rather than in a hospital. This includes either preventing unplanned hospital admissions or supporting people to safely leave the hospital sooner. There is considerable overlap between VW and hospital at home (HaH) models of care, and these terms are sometimes confused or used interchangeably [4]. Frequently used definitions state that HaH services provide face-to-face care at home through community-based multidisciplinary teams [5], whereas VWs are hospital-led and managed to enable the delivery of at-home acute care, monitoring, and treatment using a variable combination of face-to-face care and remote monitoring (eg, through apps, technology platforms, and wearable devices) [6,7]. The scope of VWs as a model of care varies from models using remote monitoring technology to predominantly operate without in-person intervention (eg, telehealth hubs with remote monitoring) to models more heavily reliant on multidisciplinary teams delivering in-person care in patient homes [8]. Therefore, the latter end of this continuum of care overlaps largely with HaH models. VW or HaH models of care have been used across various countries, including the United States [9,10], Australia [11], the Netherlands [12], and Saudi Arabia [13].

During the COVID-19 pandemic, VWs were implemented in the NHS England (NHSE) to manage some patients with COVID-19 infection through monitoring their oxygen levels at home using a portable pulse oximeter, a small device that attaches to a patient’s finger to rapidly measure blood oxygen levels. Patients recorded readings from the pulse oximeter in a diary and were instructed to call the hospital should readings fall below a given level [14]. In some hospitals, oximetry readings were recorded in an app and displayed on a dashboard that the clinical teams could access continuously via a tablet or computer [15]. Since then, VWs have been expanded to different care pathways, primarily for acute respiratory infection and people living with frailty, as per NHSE’s (a public body that oversees budget planning, delivery, and operation of commissioning of the entire NHS in England) instruction [16]. A key criterion of this instruction was that VW models should be enabled by technology, that is, the management of patients via a digital platform managed remotely by a clinical team. VWs have been adopted and introduced in different ways in both primary and secondary care settings across England. As a key part of NHSE’s plans to improve the responsiveness of emergency and urgent care and increase capacity, the national ambition was to have 40 to 50 virtual beds per 100,000 population by December 2023, the equivalent of up to 24,000 beds in total [16]. As of September 2023, the NHS had delivered 10,000 VW beds, with >240,000 patients treated in VWs [17].

Evaluations of VWs are underway. As of July 2022, evidence from reviews of randomized trials indicated that there is consistently low to moderate certainty evidence that clinical outcomes, including mortality and readmission, for patients treated in VWs are as good or better than for those treated as inpatients [4,18]. The evidence on cost-effectiveness is less clear. Although there have been many studies investigating the costs of VWs to health care providers, nearly all of these studies have methodological problems, which may mean they overestimate cost savings. For example, few studies conducted cost-effectiveness analyses, with most having cost-minimization and cost-saving designs, and many studies disregarded informal care costs to patients and carers [4]. There is low-certainty evidence from reviews of randomized trials that patient satisfaction may be improved by VWs compared to inpatient care [4].

On July 1, 2022, 42 integrated care systems (ICSs) were established across England on a statutory basis [19]. These are partnerships of organizations (eg, secondary, community, primary, social care, and mental health services) that come together to plan and deliver joined-up health and care services and to improve the lives of people who live and work in their area [19]. Each ICS has an integrated care board (ICB). The ICBs have strategic and financial responsibility for the planning and delivery of health services. Within the NHS, “commissioning” refers to the process of assessing needs, planning and prioritizing, and purchasing and monitoring health services and is undertaken by those in senior leadership roles [20]. The mandate for VWs was developed centrally by NHSE and detailed the national ambitions and instructions for ICSs to then adopt and implement in their own way according to local needs. ICS commissioners and ICB members have been tasked with developing detailed plans on how they intend to move from current practice to using technology-enabled VWs. These plans are to be based on partnerships between secondary, community, primary, and mental health services. ICS commissioners have an important role in the development of implementation plans and the effective adoption of this new model of care in clinical practice.

Effective adoption and rollout of VWs will depend upon the perceived acceptability and feasibility of this care delivery model to stakeholders. The Medical Research Council framework for developing and evaluating complex interventions [21,22] advocates the use of qualitative research to ascertain what is viewed as acceptable or unacceptable to key stakeholders involved in complex health care interventions. The use of qualitative inquiry in intervention evaluation can provide a contextualized understanding of important experiences, enablers, and constraints regarding the implementation of an intervention across a range of settings. Findings from such research can therefore provide useful insights for decision makers working across different contexts.

This qualitative study drew upon the Consolidated Framework for Implementation Research (CFIR) [23]. The use of theory is critical to understanding the likely processes through which an intervention may work [22]. However, there is an array of often-overlapping implementation theories described in the literature, making the selection of a particular theory for implementation evaluation problematic. The CFIR addresses this issue by providing an overarching, comprehensive framework, comprising common constructs from published implementation theories; the CFIR is therefore well-suited to guide the evaluation of the implementation of health care delivery interventions [24]. The updated version of the CFIR [25] was used in this study. This consists of 5 overarching domains (innovation, outer setting, inner setting, individuals, and implementation process), each with multiple constructs (eg, for the innovation domain, constructs include innovation relative advantage, innovation complexity, and innovation cost).

### Aims and Objectives

This study aimed to develop an understanding of the acceptability and feasibility of adopting and implementing VWs across ICSs in England from commissioners’ perspectives, following the issue of the VW mandate by NHSE in the April 2022. Our objectives were to identify commissioners’ views on the potential benefit of VWs within the current health care system, identify the barriers and facilitators to adoption and implementation, and ascertain the ways in which the VW model of care could be optimized in future practice.

## Methods

This study is reported in accordance with the COREQ (Consolidated Criteria for Reporting Qualitative Research) checklist (Multimedia Appendix 1) [26].

### Study Design

We conducted a qualitative study using one-to-one semistructured interviews via an online platform (Microsoft Teams).

### Sample

The sample comprised individuals working in commissioning roles in ICSs in England. In total, there are 42 ICSs that each provide health care to around 500,000 to 3 million people. Commissioners are staff in senior leadership roles who are involved in the planning of various health care services, including the purchasing of technologies and monitoring implementation of the VWs program. Purposive sampling was used to select staff from ICSs in different geographical regions and those at different stages of the adoption and implementation process to enable comparisons across sites and to identify important contextual factors affecting implementation.

### Recruitment

Potential participants were initially identified by our NHSE collaborators as being eligible to participate in the study based on their job role in NHSE. They were approached about the study, and permission to share their contact details with the research team was obtained. The research team invited them by email to participate. Snowball sampling, whereby participants were asked to identify other potentially eligible participants to invite to the study, was subsequently used. Participants were provided with an information sheet that outlined the requirements for participation and data protection measures.

### Data Collection

In-depth one-to-one semistructured interviews were conducted via Microsoft Teams by 2 members of the research team (LJM and FG). Only the researcher and participant were present during the interviews. The topic guide (Multimedia Appendix 2) used broad open-ended questions relating to the study aims rather than mapping questions directly to the CFIR. This enabled respondents to discuss issues of importance to them and encouraged a logical conversational flow to the interview, in line with published methodological guidance [13]. The topic guide was developed by the researchers with input from patient and public involvement and engagement (PPIE) representatives and NHSE collaborators. Members of the NHSE team responsible for the VWs program nationally reviewed the topic guide, and modifications were made according to their feedback. Questions were reviewed and revised during the course of data collection to explore emerging issues in more depth. Interviews were video-recorded via Teams and converted to audio-only (MP3) files for storage. Audio files were transcribed verbatim; transcripts were anonymized, ensuring all identifiable names, places, and events were removed. Data collection stopped once the research team agreed data adequacy had been achieved [27,28], which was based on the quality and sufficiency of data in terms of the richness of and variations in the data in relation to the research aims and objectives. To aid this judgment, we applied a color-code rating system to our preliminary themes from the first stage of analysis (refer to Data Analysis section): “red” signified an abundance of data with no new insights gained from subsequent interviews; “yellow” signified data were voluminous but new insights or alternative perspectives were still emerging; and “green” signified that data were minimal and more insight was needed through further data collection. Color codes were updated with each subsequent interview until all preliminary themes were coded red.

### Data Analysis

The analysis involved 2 separate stages. First, in line with rapid qualitative inquiry methods [29,30], we conducted the preliminary analysis using rapid appraisal procedure sheets. These summarized key findings after each interview based on interview field notes and were then discussed with our policy partners at NHSE. This facilitated the timely dissemination of key findings in a fast-moving policy research area.

The in-depth analysis took place in the second analysis stage. Interview transcripts were analyzed thematically and structured using the framework approach [31,32]. NVivo (version 14; Lumivero) was used to facilitate analysis. The analysis involved both inductive (ie, data driven) and deductive (ie, theory driven) processes and comprised 4 main steps. The first step was reading and rereading transcripts and listening to audio recordings, noting key ideas (to aid familiarization). The second step involved line-by-line coding of transcripts to the updated CFIR [25] (reflecting the deductive element of analysis). CFIR domains were adapted to this study, and a codebook with definitions of domains and subdomains was created. Where it was felt that data did not fit within the framework, new codes were created. In the third step, each CFIR domain was plotted onto a separate thematic matrix, with subdomains (“codes”) presented in separate columns and participants (“cases”) on individual rows. Data were summarized into cells in each matrix according to the respective case and code. In the fourth step, matrices were reviewed and thematically analyzed (reflecting the inductive element of analysis), drawing upon connections made between codes and cases and reflecting on the overall narrative in relation to the research objectives. This facilitated the determination of the final overarching themes and the key issues and meanings within those themes.

The use of a combined inductive and deductive approach in the analysis helped to ensure comprehensive coverage and theoretical consideration while retaining the narrative and nuance of traditional qualitative approaches [33]. The findings presented in this study’s results reflected the final overarching inductive themes derived from the final stage of analysis.

### Research Team Reflexivity

Interviews were conducted by 2 female researchers (both holding PhDs and employed as research associates at the time of data collection) with previous training and experience in qualitative interviewing but little prior knowledge of the VW program and no previous relationship to participants. Participants had no personal knowledge of the researchers. This reduced the likelihood of participants providing ambiguous responses relying upon existing shared understandings. Transcripts were coded and analyzed by multiple members of the research team with a variety of disciplinary backgrounds, including health psychology, sociology, behavioral science, and public health. We also included the perspectives of an NHS clinician as well as a PPIE representative within our analysis discussions to ensure that interpretations were considered from multiple perspectives and that findings were relevant to both practice and public interest.

### Ethical Considerations

This study was approved by Newcastle University’s ethics committee on July 28, 2022 (23846/2022). A consent form was emailed to potential participants before the interview, and informed consent was recorded verbally at the start of the interview. Transcripts were deidentified, with any information that could potentially identify an individual removed or replaced with vaguer descriptions to ensure participant anonymity. Participants were given a unique identification number on recruitment to the study, which has been used when reporting participant quotations in the paper. No compensation, financial or otherwise, was provided.

## Results

### Participant Characteristics and Context

A total of 20 semistructured interviews were conducted between September 2022 and January 2023 (initial implementation plans for VWs were submitted around April of 2022). No participants dropped out once recruited to the study. Interviews lasted between 30 and 80 minutes. Participants’ characteristics are shown in Table 1. There are different models of VW, and their emphasis on technology varies in line with patient needs and preferences. Descriptive information regarding the types of VWs being implemented, as described by commissioners in this study, is available in Multimedia Appendix 3.

Four overarching themes were identified reflecting the acceptability and feasibility of key adoption and implementation processes: (1) assessing the need for VWs, (2) coordinating a system approach, (3) agreement on Program Outcomes: NHSE Versus Organizational Goals, and (4) planning and adapting services.

Overarching themes and subthemes are presented in Textbox 1.

**Table 1:** Participant characteristics (N=20).

Characteristics			Participants
Age (y), median (IQR)			48 (34-60)
**Sex, n (%)**			
	Female	16 (64)	
	Male	4 (36)	
**Professional role, n (%)**			
	Executive or director	6 (30)	
	Program lead or manager	6 (30)	
	Senior manager	6 (30)	
	Clinical lead	2 (10)	
**Employer, n (%)**			
	NHS^a^ ICS^b^	5 (25)	
	NHS Trust	12 (60)	
	AHSN^c^	5 (25)	
**Geographic location, n (%)**			
	North East and Yorkshire	12 (60)	
	North West	4 (20)	
	Midlands	1 (5)	
	South West	3 (15)	

^a^NHS: National Health Service.

^b^ICS: integrated care system.

^c^AHSN: Academic Health Sciences Network.

**Overarching themes and subthemes.**

**Assessing the need for virtual wards**

- Tension for change
- Replacing hospital beds versus additionality of service
- Need for technology enablement

**Coordinating a system approach**

- The translation of instruction: definitions, language, and terminology
- Partnerships and connections

**Program outcomes: National Health Service England versus organizational goals**

- Targets and evaluation
- Timelines

**Planning and adapting services**

- Standardization or flexibility
- Identifying interoperable technology “solution”
- Receptivity and need assessments of patients and staff

### Assessing the Need for VWs

#### Tension for Change

Many participants expressed a need for change to the ways in which care is provided within the NHS to counter the high levels of unmet demand for hospital beds and clinician time:

The pressure in secondary care is unbearable...the whole system feels just about broken. And if we carry on doing things the way we’ve done them then we’re going to get what we’ve always got and we’ve got to do something different.

P05

Increasing capability and capacity in community care services to mitigate pressures in hospitals was a key strategic system-level aim with which VWs aligned. VWs also aligned with commissioners’ aspirations for the NHS to provide quality patient-centered care and consequently were consistently reported by commissioners to be the “right thing to do:”

I’m a passionate believer in community-based care. We hospitalise people far, far too often when we can wrap services around people in their own home and keep them in a safe place. So, it’s absolutely the right thing to do.

P01

However, there was some concern around the timing of the NHSE VWs instruction to ICSs and whether implementing a service requiring high levels of system-level transformation was appropriate given the infancy of ICSs and the recent pressures of the COVID-19 pandemic. A perceived weariness within the secondary care workforce appeared to lead to a level of despondency among some staff members and negatively impacted the timeliness of delivery of VW implementation plans:

Throwing something new in at the time at which an already busy corporate powerhouse is churning to deliver the existing asks. To then get support with something new that they don’t understand, that has come completely leftfield, that’s got money attached to it, but only if you respond within five minutes, and you’ve got to plan your workforce, it’s a big ask.

P08

Some of the implementation plans for last year weren’t delivering. Now that probably is a consequence of COVID, so we had to support and put in mitigation for supporting them to actually achieve what they could achieve....All really struggled last year with still coming out of COVID.

P03

#### Replacing Hospital Beds Versus Additionality of Service

There was variation in the extent to which participants believed that VWs would reduce the number of patients receiving care in hospitals. It appeared that many participants interpreted the NHSE instruction to mean that referral of patients to VWs would reduce the number of hospital beds required and that costs saved through this reduction in hospital beds (ie, reduced staff resources) could help to provide longer-term funding for VWs. Though some were optimistic that having more patients on VW beds would mean fewer patients cared for in hospitals, many felt that the existing unmet demand for hospital care inevitably meant that the hospital beds “released” by VWs would be taken by someone else:

There’s this simplistic thought that this person would be in that hospital bed, and now they’re in their own bed, so we’ve then saved that bed. And again, we also know that’s not true, because that bed will be used for someone else.

P18

In this respect, some participants reflected that it was likely that VWs would always be an addition to the health care system:

In real terms, it is about additionality...it’s not like we’re saying we’re closing a hospital ward to fund this. We’re saying that we’re still going to have a hospital ward open...I think what it will do is provide additional [care delivery] capacity.

P19

Some felt that having this additionality of service could improve the flow of patients through the system:

For the system, it will hopefully improve patient flow out of hospital. It’ll hopefully impact on people being presented at emergency departments or being admitted to hospital. If you can contain somebody at home, manage their acute episode, put the right care in for a certain period of time, and then reduce that.

P07

The perception that VWs would unlikely lead to the closing of hospital beds inevitably meant that participants questioned the potential for long-term cost savings:

I think it could only be cost saving...if you could close hospital beds...But I can’t see that that’s possible.

P06

However, the lack of cost-saving potential was not equated to the lack of success of the VW program, with clinical outcomes typically considered a priority:

Clinical success does not equal financial saving...This will be an additional cost to the system.

P02

#### Need for Technology Enablement

The need to improve and expand community-driven care was widely accepted among commissioners, but there was less consensus about the need to incorporate technology as part of the VW service offer. While NHSE recognizes that there are “different models of VWs, and their emphasis on technology varies in line with patient needs and preferences,” the NHSE guidance with regard to the use of technology, published in April 2022, states: “Technology enablement means the management of patients via a digital platform managed remotely by a clinical team” [34]. This may include patients measuring and recording vital signs in an app or continuous monitoring of vital signs via a worn device, which reports to a clinician dashboard, with alerts sent when readings fall outside agreed parameters. Participants reflected on the limited evidence for the advantages of using technology, particularly to remotely monitor patients, and some felt this led to uncertainty and nervousness among clinical staff members and resistance to change, which hindered engagement with the instruction. As such, the technology element of the VW implementation plans was typically less developed than other aspects of the program:

You haven’t got a machine in front of you, you’re going on what the remote monitoring devices are telling you. You can’t sit and play with a device. You’re not sure that that’s giving you the right recording.

P03

I think there’s a bit of a nervousness and a bit of a reluctance to maybe see the benefit of tech...I think it’s sometimes easier to revert to what you know, and what you feel comfortable with.

P13

Several participants expressed reservations about the extent to which technology could improve or enhance care provision in the community. This was particularly common among participants in areas where existing services do not currently involve digital technology. One participant who was working to make existing HaH services technology enabled to meet the NHSE definition reported:

[NHSE] have extrapolated that model into a broader virtual ward model, and I don’t think the digital monitoring is the be-all and end-all...frightened breathless patients want somebody at the end of the phone...somebody who can come and see them and...sort them out if they need urgent intervention.

P05

Furthermore, the mandate for VWs to be enabled by technology appeared to instill an illogical approach of identifying technology solutions first and attempting to develop a pathway around this, rather than assessing the pathway needs first, and then identifying how technology may facilitate and enhance these needs:

[VWs should be based] on a clear clinical need and solving a problem that is already identified as existing, as opposed to creating a problem to solve it.

P16

I care about the patients, the care we deliver. And if I believe that adding tech will add value then I’ll do it. But I won’t do it just to get my numbers up, because it’s flawed.

P05

This was exemplified in an area where various technology solutions had been trialed and implemented in previous projects with the support of an Academic Health Science Network:

Those were definitely the most successful, where there was already a need and we were able to facilitate that...The less successful ones were the ones where services were approached and offered it...Where they hadn’t already identified that that might be a thing that they want to do...it was trying to shoehorn technology into a pathway where either they weren’t ready [or] they didn’t really see a need.

P16

This highlighted the importance of enabling local places to assess and identify the specific needs of their respective care pathways.

### Coordinating a System Approach

#### Translation of Instruction: Definitions, Language, and Terminology

VW service provision involves both “top-down” instruction from NHSE and “bottom-up” innovation by clinicians in practice. Commissioners were typically responsible for translating the NHSE instruction to care providers and other organizations in their system locally.

There was apprehension among commissioners about whether organizations could successfully come together to deliver the service, given that different sectors had contrasting interpretations of the VW definition and what patients receiving care at home meant:

I know there will be different views of what a patient receiving care at home means to people...traditionally that would mean that that patient is under a GP’s care, perhaps, and it is more of an outpatient environment.

P04

The difficulty in the translation of the instruction appeared to be compounded by the use of hospital-centric language in the NHSE mandate. Several participants reflected that the terminology used to describe VWs was confusing, stemming from an attempt to apply “acute care language” to community services:

If we’re wanting to manage people in their own home...we need to stop calling them “beds,” we need to be talking about caseload instead...the notion that you would have an empty virtual ward bed doesn’t make sense.

P18

Indeed, some felt the term “VWs” was problematic in itself and equated the model too closely to hospital care rather than placing emphasis on community care delivery:

“Virtual wards” itself is quite a problematic term...we are very much a community-led model. “Virtual ward” can make it sound...that you’re going to receive exactly the same as what you received when you were at hospital.

P14

These reflections highlighted a “hospital-centric” view of the VW service, which may be problematic given that VW models involve the broader health care system, so they could, therefore, impede coordinating a system-level approach to the service. Alternative terms used by a clinician, such as *extra bed days at home* rather than *early discharge* and *alternative to admission* rather than *admission avoidance* [P18], were reportedly preferable.

Participants agreed that each locality would need clear communications to patients and staff about what their VW model offer is and is not and how the service differs from other care pathways available to them, such as being an outpatient. This was challenging for ICS staff to develop centrally as the offer varies across and between trusts. One participant reported that their ICS had reached their own consensus on the scope of their VWs but asserted that there should be a consensus on a national level:

I think the national definitions are confusing...If the care at home is replacing an acute admission and there’s a treatment plan and the right level of oversight, and we can be assured that if that virtual ward package of support wasn’t available, the person would have to be in hospital, then that should count as a virtual ward patient. That’s the consensus for you in our ICS. So, I think that needs further debate nationally.

P06

This perceived lack of consensus was alluded to by other participants:

At the moment we’re building a new model of care with no clear understanding.

P17

In this respect, it seems more clarity is needed from NHSE to ensure a shared consensus on what constitutes a VW for all stakeholders to facilitate a system approach to the VW program.

#### Partnerships and Connections

Most partnerships and connections that supported the implementation of VWs were local to place, either across or within ICSs. These connections ranged from forming a network of foundation trusts (NHS organizations demonstrating the highest clinical standards, quality leadership, and record of patient responsiveness and safety) within the ICS to an individual clinician peer-to-peer model of sharing. Primarily, their function was to share learning, best practices, and achievements:

What are we doing across our two acute trusts is, learning and sharing across the two. So [trust name] sharing a lot of their ideas around their multi-specialty pathways, because they have actually got a well-established pathway. Then actually, the other trust sharing across with [trust name] as well. It’s very much, a sort of, sharing and building kind of model.

P13

Some commissioners discussed the usefulness of having a local clinical lead for the VW program who shared knowledge across other ICSs in their region. In particular, having empathy for the challenges associated with the development and implementation of VWs was viewed as important. This suggested the importance of having local VW “champions” who can positively drive the enthusiasm and development of VWs at a regional level:

We have a clinical lead at region who is also working on implementing it in her own system, and she has been fantastic. So she has come and talked to our teams about what they’re already doing where they are. But she’s also, because she’s living and breathing it, when I tell her about the challenges, she recognises that that is a genuine challenge, and not saying, “Yes, but why can’t you just do it?”

P18

Again, at a local level, 1 ICS had help from a commissioning support unit in their region, which facilitated VW implementation. They provided project management support in developing the VW model, with plans and tasks to complete to ensure they met the delivery timescales and national deadlines:

That’s been really quite helpful because they’ve been almost like a go between, between us and NHSE if you like, and helping us to develop the plans and things that we need to do in terms of meeting timescales for delivering. Keeping us abreast with everything that’s been required nationally so that we don’t miss a deadline, or we make sure everything’s been delivered that we’re meant to.

P13

Participants described having convened “multiorganizational” steering groups comprising relevant representatives from across the system to support the development of VW service provision. It was reported that the design of the VW care delivery model was largely led by clinicians in acute trusts. This may be due to clinicians having ultimate clinical responsibility for patients in VWs, leading to resistance to delegate to community staff they do not know or trust. However, participants acknowledged that having the service designed largely by acute staff was problematic, as acute clinicians do not necessarily understand the community care “world,” particularly with respect to how technology may serve to support patients in their own homes. This was discussed in terms of risk aversion, with community staff perceived as being less risk averse than staff in acute environments.

Community staff will have a different view. Because they’re [services are] run with a bigger risk...there’s lots of people who are not well who live in the community, who never hit hospital, who are well cared for. So, they’ll probably have perhaps more confidence around using remote digital technology.

P07

This highlighted the importance of developing and establishing ways of connecting health care staff from traditionally distinct care delivery settings and supporting them to share their knowledge and experience.

### Program Outcomes: NHSE Versus Organizational Goals

#### Targets and Evaluation

Participants reported that, as commissioners, they had a key role in ensuring the targets set by NHSE were met and reporting and monitoring procedures were followed. However, there were tensions between the reporting requirements of NHSE and what commissioners believed were important indicators of success. Many felt that there was too much focus on the number of beds saved at the expense of other measures of success, for example, reduced waiting times:

Let’s not get too caught up in, how many beds have we saved, let’s think about what all of the other measures are that we’re trying to achieve,...length of stay,...patient experience,...clinician experience.”

P18

Concerns were expressed about the “short-termism” of the monitoring and evaluation measures in place that were perceived as *“*setting ourselves up to fail*”* (P15):

I think we’re so politically driven to, on a few metrics...we don’t necessarily think about this in a long-term perspective, i.e. we don’t think about some of the big quality markers that really represent value in the long-term for people and for systems.

P15

It was clear that the most important markers of success for health care providers were patient experience and outcomes, as opposed to the hard metrics put forward by the national team. This was due to participants being driven by patient-centered care, often cited as a key motivator and driver of behavior:

That willingness to put the patient at the centre of what we’re doing, and not the service that you work for, and not the organisation that you work for, or not the criteria that’s in front of you, but the person in front of you.

P08

Other measures of success proposed by participants included patient empowerment and autonomy:

I think you empower people, you measure some quality if people feel supported, people feel care’s coordinated. And they’re some of the big markers of people not wanting or ending up in hospital or dying where they want to die.

P15

#### Timelines

The timescale for implementation was considered too short, with participants perceiving the 2-year funding period to be inadequate to evidence outcomes and evaluate the efficacy of the VW program:

We won’t have enough evidence to say, “Look, it’s working, let’s carry on with this,” because you won’t have had enough time to do that.

P14

Two years to transform a system was not considered viable, regardless of the funding available. Many held the view that NHSE should commit funding to enable sites to run VWs for longer and obtain proof of concept, even if this meant less funding in the first instance:

It’s unrealistic to think that this level of change can be switched on really, really quickly. And I’m yet to see any system, unless they were already on that path, who said, “Oh yes, because we’ve suddenly got all this money, then we find it really easy to transform services.”

P18

The way we do these things needs to be much more sustainably thought through...starting small and incrementally building up with funding over a longer term would be the way I would always say we should try these things rather than full-scale, two-year implementation, no more money in year three.

P15

However, there was acknowledgment that uncertainty over funding across health and social care on a national level would make a prolonged funding model unfeasible:

I think the problem is the political element to this and how do you have the ongoing funding over five years when there’s so much political uncertainty around funding in the NHS and social care.

P15

Without evidence of the impact of VWs, some believed it would be difficult to secure continued funding:

So in terms of sustainability, systems are expected to then find that [funding] within their own financial envelope. So it’s really quite crucial that we can demonstrate an impact to both patients and cost.

P19

There was concern that the lack of time investment given to the VW initiative could lead to the national team overlooking something that is promising but is not being given the required time to provide evidence of real success:

I think clinicians do think that this is a way forward, and this is where we should be heading. But by trying to do it too quickly, with ridiculous punitive measures in around success, I think we could throw the baby out with the bathwater.

P18

### Planning and Adapting Services

#### Standardization or Flexibility

The various VW models described by participants were all highly complex, requiring multiple members of staff across different organizations working together to deliver care. Where existing community or HaH services were in place, commissioners reported being involved in shaping service provision less frequently, appearing to give trusts more autonomy to adapt or develop their own pathways to meet the VW definition. However, commissioners recognized that this was leading to variability across the system.

Participants in places where services were less developed reported a key part of their role as commissioners was to support delivery planning teams to develop standard operating procedures (SOPs) and processes for VW models:

We have to do a lot of influencing and coaching around what a design could look like. Working with business analysts to map out requirements of the workstreams—to understand the current situation and “future state.”

P17

While many participants reported that local delivery teams were best placed to develop the VW care pathway, some systems had made a conscious effort to create a standardized delivery model and adapt SOPs from other trusts. They were mindful that the templates would be adapted by the trusts to meet their specific needs but thought that this would bring some level of consistency in terms of service provision and equity of access:

We want to standardise delivery as much as possible so that the patient access is equitable across the system.

P06

Participants identified several important planning activities that were needed to provide care to patients in the community successfully and monitor them remotely, including identifying roles and responsibilities of clinical staff for the duration of patient care, defining patient eligibility criteria and how that is applied, clarifying the need for remote monitoring (frequency and modality), and creating a shared patient record accessible across the partnership organizations.

Several participants expressed the notion that once these key processes had been conducted, the same design approach could be applied by colleagues to manage patients with other conditions as most governance, IT requirements, and processes could be replicated:

Risks will need to be considered and tailored to the new conditions that they’re managing but the principles and the process, a lot of it will be the same.

P09

Providing general guidance about what implementation processes and considerations are needed may be helpful in ensuring that the VW services offered are of a similar care quality standard.

#### Identifying Interoperable Technology “Solution”

Incorporating the “technology-enabled” element of the VW mandate was challenging for those with existing VW services that were not technology-enabled. They reported having to develop a digital strategy and adhere to the strict information governance arrangements to ensure that the technology met requirements. One participant reported that they already had such processes completed for a prior service before the VW mandate was announced but commented on the length of time it had taken and noted this as a reason some areas were considerably further behind others in their VW implementation:

Things such as the right IG protocols in place, not having e-prescribing programmes in place, not having after care testing in place, etc., not having the shared care record in place. We had to do all of that, and that took us at least 18 months anyway, so we’re ahead of the game.

P01

Participants described difficulties that teams had faced when attempting to integrate existing remote monitoring devices, procured through previous projects or initiatives, into the VW services they were developing. For example, several participants reported interoperability difficulties, requiring a need for the dashboard to integrate with the patient medical records. They reflected this was an issue that was broader than the VW program:

At the moment...we’ve got 19 different sign-ins for a patient...the same problems that we’re hearing with acutes [team]...if we want to really mitigate risk around digital disconnect...we need to make a better online experience.

P17

Interoperability of the technology in terms of having shared electronic patient records (EPRs) that all care teams can access in real time was considered critical to ensuring safe services. One participant described a “complete patchwork quilt of tech” (P06) across ICSs, reporting that EPRs are not in place in some organizations. This was considered a wider issue across the NHS but was deemed to be a particular obstruction to the delivery of VWs:

The current technology profile across our system does not make the delivery of virtual care as easy as it could be. In fact, for some staff it makes it harder...It’s an issue for the NHS full stop, but I think it’s a real issue for virtual wards.

P06

Interoperability was also a key issue in terms of EPRs for medication prescriptions. One participant noted a near miss with regard to relative contraindication due to general practitioners not having access to VW patient prescribing data:

We did have a near miss where...the patient had been off to the doctor for something else and now was changing their medication. There would have been a contraindication with their virtual ward medication. Luckily, it was picked up. Nothing happened, but obviously, it alerted us to the fact that we needed to make sure we got the EPR so that the GPs could know.

P20

There was also added complexity as it was deemed important that appropriate information about a patient is shared, where relevant, with the local authority regarding social care and housing arrangements to allow tracing of patients being admitted and discharged from VWs. These data could help to monitor the effectiveness of VWs along with any implications for health inequalities.

#### Receptivity and Need Assessments of Patients, Carers, and Staff

Although patient and carer need assessments and prospective receptivity to VWs were not typically conducted, there was consensus among commissioners on the patient-level benefits of VWs (eg, being more comfortable, having social support networks around them, less risk of hospital-acquired infection, and being able to sleep and eat better). However, it was acknowledged that VW care models would not be appropriate for all patients due to inadequate home environments, rising costs of living, and digital exclusion and participation issues:

There’s a very small niche of this absolutely tech enabled person, who’s got a smartphone, who’s onboarded to tech...got a warm house...got enough food to eat to keep them well...where they can afford to plug in the [implement] and afford the electricity.

P01

There is a risk that this type of care might only be accessible if you are already digitally enabled...and you’re not poor.

P06

Participants reported clinical teams were still in the process of planning assessment criteria for patient suitability for VWs. Nevertheless, participants reflected upon the importance of patients being able to make an informed choice about whether to be referred to a VW:

Is the patient comfortable with being on a virtual ward as opposed to being on a hospital ward? And that they should have that choice. They might, kind of, fit the definition of the patient cohort that would be appropriate for being on a virtual ward, but are they okay with that?

P16

Some VW models required patients to self-test and input their data into an app or a paper diary. This was regarded positively, affording a more “personal approach” to care and giving patients greater autonomy and responsibility for their own care:

[Patients] can see then whether their condition is improving or whether it’s deteriorating, and they can see what action is going to be taken as part of that.

P10

However, there was also concern that self-monitoring adherence may decline over time (“people that started to use the app but, for some reason, have tailed off and stopped;” P11). Thus, further exploration, evidence, and guidance may be required as to what remote monitoring technologies are appropriate for which patient groups and conditions.

Participants also acknowledged the impact of VWs on patient carers, with increased burden identified as a risk in terms of increased responsibility, lack of respite, and potential financial issues:

Because otherwise, their loved one would be in hospital having care, and now they’re at home, an increased carer stress, and a feeling of additional responsibility...There may be an economic burden for carers that we don’t fully understand, if they have the take more time off work.

P06

Carers who are actually caring for that individual when somebody goes and hospital for a few days, actually gives them a little bit of a break.

P13

By contrast, some positive feedback was reportedly received from carers, relating to having a greater understanding and input into the care the patient receives:

If their loved one was in hospital...they felt a bit out of the loop and they weren’t really sure what was happening or what to expect when the person they cared for came home. Whereas now, they feel part of the conversation.

P19

Therefore, it seems imperative that carer perspectives are also built into the design of VW services:

Something that we definitely want to do is reach out and have conversations with carers as well as well as people who would access this service to get that understanding.

P13

With respect to staff, VW delivery teams (eg, community, acute, telemedicine, urgent care, and out-of-hours teams) needed to have a clear understanding of the service offer and areas of responsibility across pathways and partnerships. One participant reflected on their experience of working with care home staff and emphasized the importance of conducting a skills mapping exercise before introducing technology to understand the training needs of those delivering the program:

*If we were to do work again in care homes or the care sector, we’d spend a lot more time upfront doing skills analysis, digital literacy analysis,like whether they’re ready for different digital pathways.* [P16]

In terms of staff receptivity, participants believed the VWs were more successful in areas where they were led by clinical enthusiasts who had an overwhelming conviction in the VW model:

It’s having people...who are willing to try things, to really step up and put their head above the parapet and say, “Do you know what? I know we’ve been doing it this way for 30 years, but this is better.” It’s that investment in your clinical leadership that you need, to make this change.

P02

Indeed, 1 participant noted that their approach was driven through the deliberate identification of clinical enthusiasts in the first instance:

So that was our approach really, is go where the enthusiasm is, that those who are keen to do it and, actually, let the rest of the system see the benefits.

P19

However, participants reported that enthusiasm for VWs varied among clinicians. Resistance to change among clinical staff appeared to be a barrier to the willingness to adopt VWs, with lower receptivity to VWs perceived to be more common in staff who had worked in the system for longer and were used to caring for patients in more traditional ways:

One of the consultants who’s really quite dead set against virtual wards is a consultant that’s been in the system for quite some time...is [a] strictly traditional consultant [in] the way that he has traditionally managed that work...whereas one of our most enthusiastic consultants is a younger person that hasn’t been around for quite so long.

P19

Participants also described some clinicians as being nervous about patient safety. Clinicians were apprehensive that VWs diminished opportunities to observe patient symptoms and expressions that may provide additional information to determine clinical decisions:

In someone’s home, there is no observation. So often you might need to work more with the patient’s carer and family to make sure that they are looking out for some of the idiosyncrasies that you may, or may not, think are important.

P03

The technology and digital literacy of staff were also highlighted as factors influencing the adoption and implementation of VW services. For some, the VW program was the first time they had used digital devices, and the use of technology was considered a steep learning curve:

This was their first attempt at doing remote working, remote monitoring, remote consultations...in some cases they were still quite new to digital at all. So...to then go, “Okay, well we’re going to completely change the way you work and bring in this new technology.” It was quite a steep learning curve.

P16

However, it was felt that adequate training of staff on new technologies mitigated initial apprehension and concern over using technology in VW services:

So I think the clinicians [are] finding it [technology] really straightforward and easy...I think there’s that little bit of concern and worry prior, but once the training has all happened, they feel then confident with it.

P20

There were also reports of fear and uncertainty among clinicians regarding liability in the case of adverse events and where the ultimate responsibility for VW patients lay, *“*There is a fear of medico-legal litigation, if things go wrong*”* (P03). Clarification of these issues was considered an important prerequisite to addressing these concerns and improving engagement.

Furthermore, the limited capacity of clinical staff to plan through the care pathway was highlighted as a hindrance to the development of VW delivery plans:

The workforce, within the acute trusts at a consultant level, is minimal...they just don’t have the capacity...taking medical oversight and ownership of [virtual wards], is an additionality to them. It’s easier for them to continue in the way that things always have been, without seeing the bigger benefits.

P12

## Discussion

### Principal Findings

This qualitative study analyzed commissioners’ perspectives on the adoption and implementation of VWs in ICSs in England. Findings demonstrated a strong desire for system-level change in care provision. Though commissioners had an enthusiasm for the VW program as a means to improve patient-centered care, there were concerns about the timing of the mandate and uncertainty over the extent to which hospital beds could be closed to release funds for VWs or whether their purpose is to free bed capacity for those more in need of in-person care. There was also uncertainty over the need to incorporate technology into the VW offer. The VW mandate involved convening a system approach to implementation; however, this was hindered by differing interpretations of the instruction due to a lack of clarity in definitions within the national mandate and the use of hospital-centric language. Other key perceived challenges to implementation included narrow parameters of success, unrealistic timescales to provide evidence of effectiveness the lack of interoperability of technology and time-consuming procurement procedures, and liability concerns. Commissioners felt VWs could offer numerous patient-level benefits but emphasized the need to assess the suitability of patients for the service. Successful implementation was considered contingent upon having motivated and passionate clinical leads.

To our knowledge, no other study has analyzed perspectives on the adoption and implementation of VWs specific to the national 2022 NHSE mandate. However, some of our findings can be understood in the context of research on previous VW and HaH care delivery models. Similar to this study, reviews [4,35] identified patient-level characteristics (eg, technology access, technology literacy, and appropriate home environments) as important issues and emphasized the need for careful consideration of patients’ needs and characteristics before enrollment in VWs. Other studies [36] have highlighted the importance of convening multidisciplinary teams and ensuring integrated involvement of both primary care and hospital staff in VW models. This is in line with our findings on the importance of having multiorganizational steering groups, with particular emphasis on connecting acute care and community care sectors. Furthermore, a recent review on the use of technology in telemedicine [37] identified common barriers to implementation, including resistance to change, technology-challenged staff, interoperability, digital exclusion of patients, clinician perceptions of impersonal care, and issues relating to legal liability. These barriers link to our findings regarding the receptivity and needs of patients and staff (resistance to adapt to new ways of providing care, digital literacy of patients and staff, clinician concern over lack of in-person observation of patients, and liability concerns) and identification of an interoperable technology solution. Therefore, it appears these are common barriers across technology-enabled virtual care that need addressing in future models and iterations of VWs.

### Strengths and Limitations

Strengths of this study include the involvement of multiple stakeholders in the design and analysis of the research. In addition to academic researchers, we had input from policy, clinical, and PPIE representatives at all stages of the research, including consultation on the topic guide and involvement from the early stages of analysis, to assist with the interpretation of findings and identification of key issues to further probe during data collection. Such involvement helped to ensure the rigor and trustworthiness of the data collected and the diversity in interpretation and relevance of our findings. More broadly, our collaboration with policy makers ensured that this work is policy-relevant, with a direct route to impact decision-making. This was facilitated by our use of rapid appraisal procedure sheets as per the rapid qualitative inquiry approach, ensuring key findings were disseminated quickly in such a fast-moving policy research area.

A further strength of this study is the use of an implementation framework (the CFIR). This helped to guide the analysis and facilitated the comprehensive identification of issues related to implementation. However, our use of inductive analytical techniques following initial coding to the framework ensured that the overall narrative, context, and nuance were retained in our findings [33].

There are also various limitations of this study. First, there was an overrepresentation of ICSs from northern parts of England, particularly the North East and Yorkshire. We attempted to mitigate this by asking our NHSE collaborators to identify commissioners involved in the VW program from southern geographic areas who would be willing to participate; however, our response rate for this was low. This may have been due to the limited capacity of staff in such areas. The overrepresentation of participants from northern ICSs in England may have influenced our findings in terms of the infrastructure and financial circumstances of respective ICSs and the wider social and economic circumstances of areas in northern compared to southern regions. Indeed, there is a worsening divide in terms of health care and health inequality between northern and southern England, with northern areas demonstrating lower life expectancy, higher infant mortality, and worse health and well-being [38]. In light of this, there may well be different challenges to the implementation of VWs in northern England compared to southern regions, such as the impact of the cost of living and patient suitability in terms of access to technology, digital literacy, and having appropriate home environments. A second limitation is that we were unable to characterize systematically the implementation stages of each ICS represented in the study. These data could have helped to identify more nuance in implementation issues across ICSs.

### Implications and Future Research

There are several implications of this research. First, if VWs are to be implemented effectively, there is a need to develop clearer communication strategies between NHSE and ICS leads about the purposes and intended goals of the program. In particular, uncertainty about whether VWs are intended as an additional service to increase care delivery capacity or intended solely to improve the quality of patient-centered care needs to be addressed. This may be achieved through the development of a shared logic model providing clear definitions of what does and does not constitute a VW, how VW models could reduce hospital beds, as well as detailing the potential sustainability of such models in terms of continued funding. Such communications should avoid using “hospital-centric” language and terminology to prevent misinterpretation of VWs as an acute care service, with more focus needed on the community-care aspect. Importantly, a shared consensus needs to be reached on the intended outcomes and success measures of the VW program, including patient-level criteria. Clarification and consensus around these issues could help garner support among operational staff and clinicians—strategic leadership and implementers need to push the same agenda to foster a climate where stakeholders are highly motivated and open to changing their way of working.

In terms of the technology enablement of VWs, there should be further clarification over what types of technology would be suitable for particular pathways, with examples given of how such technologies may enhance care within the pathway. This should mitigate the concern over developing pathways around technology solutions rather than assessing the pathway needs first and then identifying how technology may facilitate and enhance these needs. Providing existing and emerging evidence on the benefits and possibilities of using technology in care provision, particularly with respect to digitally enabled remote monitoring, via a national platform would also help alleviate clinician concern and trust issues regarding technologies, and this increased confidence in technology enablement could help to ease clinicians’ resistance to change.

Furthermore, need assessments of both patients and staff should be conducted. Behavioral and skills analysis of different patient populations (eg, respiratory and frailty) should be conducted to develop an understanding of training and support requirements, particularly concerning the use of technology. This could inform the creation of a standardized pro forma to identify suitable patients for a VW. A behavioral and skill analysis of those involved in the implementation of VWs would also be useful to help understand the key barriers and facilitators to implementation tasks and procedures and could inform any required intervention to enable successful implementation. Skill analysis relating to the use of technology and digital literacy would be particularly pertinent and could enable the identification of appropriate training and resources, along with the most suitable type of technology enablement for VW programs. Such behavioral and skill analysis should be grounded in a behavioral framework to identify the most relevant behavior change constructs for each stakeholder group.

In addition, co-design approaches in the design of future iterations of VW models could ensure representation from both acute and community care teams, as well as patient and carer representatives. This could improve the acceptability of VWs to all stakeholders and may help to establish a tolerable level of clinical risk with clear escalation procedures in the case of deterioration in the health of patients.

Further research is also needed to identify and describe the various models of VW currently being implemented across ICSs within the NHS. Such work could enable the development of SOPs that could be distributed nationally and adapted at the local level. Appraisal of the various models could help to identify the most effective model components and could also help to establish the costs involved and predict any potential for future cost savings compared to usual care. Specifically, evidence regarding the effectiveness and cost-effectiveness of the technology components could help to determine the appropriateness and necessity of technology enablement within VW services. Finally, more qualitative research is needed to evaluate the implementation of VWs from staff perspectives as well as patient experiences of being treated in a VW. Such research can feed into future iterations of VW service design and development processes.

### Conclusions

This study showed that commissioners involved in the adoption and implementation of VWs in ICSs in England perceived VWs to have the potential to reform some aspects of patient-centered care for some patients. However, for the model to work, there needs to be more clarity over exactly what constitutes a VW, and there should be less use of hospital-centric language in communication strategies. Furthermore, there is a need to include more focus on patient-centered measures of success, for example, clinical safety measures, measures of clinical effectiveness (patient health outcomes), and care quality (patient satisfaction), and sufficient time should be allowed to evidence these. Evidence of patient satisfaction and clinical effectiveness could help to secure future funding, enabling the sustainability of the VW model of care.

## Appendix

Multimedia Appendix 1 COREQ (Consolidated Criteria for Reporting Qualitative Research) checklist.

Multimedia Appendix 2 Topic guide.

Multimedia Appendix 3 Description of virtual wards.

## Abbreviations

CFIR: Consolidated Framework for Implementation Research
COREQ: Consolidated Criteria for Reporting Qualitative Research
EPR: electronic patient record
HaH: hospital at home
ICB: integrated care board
ICS: integrated care system
NHS: National Health Service
NHSE: National Health Service England
PPIE: patient and public involvement and engagement
SOP: standard operating procedure
VW: virtual ward
