# Supplementary material for: The Views and Experiences of Integrated Care System Commissioners About the Adoption and Implementation of Virtual Wards in England: Qualitative Exploration Study
Source: J Med Internet Res. 2024 Nov 27;26:e56494. doi: 10.2196/56494 (PMC11635316; doi:10.2196/56494)
Supplement: Multimedia Appendix 1 [file jmir_v26i1e56494_app1.docx]

Appendix 3

Consolidated criteria for reporting qualitative research (COREQ): a 32-item checklist

Developed from:

Tong A, Sainsbury P, Craig J. Consolidated criteria for reporting qualitative research (COREQ): A 32-item checklist for interviews and focus groups. *Int J Qual Heal Care*. 2007;19(6):349-357. doi:10.1093/intqhc/mzm042

| **No Item** | **Guide questions/description** | **Page No** |
| --- | --- | --- |
| **Domain 1: Research team and reflexivity** | | |
| Personal Characteristics | | |
| 1. Interviewer/facilitator | Which author/s conducted the interview or focus group? | P6 data collection sub-heading of methods |
| 1. Credentials | What were the researcher’s credentials? E.g. PhD, MD | P7 research team and reflexivity sub-heading of methods |
| 1. Occupation | What was their occupation at the time of the study? | P7 research team and reflexivity sub-heading of methods |
| 1. Gender | Was the researcher male or female? | P7 research team and reflexivity sub-heading of methods |
| 1. Experience and training | What experience or training did the researcher have? | P7 research team and reflexivity sub-heading of methods |
| Relationship with participants | | |
| 1. Relationship established | Was a relationship established prior to study commencement? | P7 research team and reflexivity sub-heading of methods |
| 1. Participant knowledge of the interviewer | What did the participants know about the researcher? e.g. personal goals, reasons for doing the  research | P7 research team and reflexivity sub-heading of methods |
| 1. Interviewer characteristics | What characteristics were reported about the interviewer/facilitator? e.g. Bias, assumptions, reasons and interests in the research topic | P7 research team and reflexivity sub-heading of methods |
| **Domain 2: study design** | | |
| Theoretical framework | | |
| 1. Methodological orientation and Theory | What methodological orientation was stated to underpin the study? e.g. grounded theory, discourse analysis, ethnography, phenomenology, content analysis | P7 data analysis sub-heading of methods |
| Participant selection | | |
| 1. Sampling | How were participants selected? e.g. purposive, convenience, consecutive, snowball | P6 recruitment sub-heading of methods |
| 1. Method of approach | How were participants approached? e.g. face-to-face, telephone, mail, email | P6 recruitment sub-heading of methods |
| 1. Sample size | How many participants were in the study? | P8 participant characteristics and context sub-heading of Results |
| 1. Non-participation | How many people refused to participate or dropped out? Reasons? | P8 participant characteristics and context sub-heading of Results (only those who expressed willingness to participate were included in the study, therefore no one refused) |
| Setting | | |
| 1. Setting of data collection | Where was the data collected? e.g. home, clinic, workplace | P6 data collection sub-heading of methods |
| 1. Presence of non-participants | Was anyone else present besides the participants and researchers? | P6 data collection sub-heading of methods |
| 1. Description of sample | What are the important characteristics of the sample? e.g. demographic data, date | P8  Table 1. Participant characteristics |
| Data collection | | |
| 1. Interview guide | Were questions, prompts, guides provided by the authors? Was it pilot tested? | P6 data collection sub-heading of methods |
| 1. Repeat interviews | Were repeat interviews carried out? If yes, how many? | No |
| 1. Audio/visual recording | Did the research use audio or visual recording to collect the data? | P6 data collection sub-heading of methods |
| 1. Field notes | Were field notes made during and/or after the interview or focus group? | P7 data analysis sub-heading of methods |
| 1. Duration | What was the duration of the interviews or focus group? | P8 participant characteristics and context sub-heading of results |
| 1. Data saturation | Was data saturation discussed? | P6 data collection sub-heading of methods |
| 1. Transcripts returned | Were transcripts returned to participants for comment and/or correction? | No |
| **Domain 3: analysis and findings** | | |
| Data analysis | | |
| 1. Number of data coders | How many data coders coded the data? | P7 research team and reflexivity sub-heading of methods |
| 1. Description of the coding tree | Did authors provide a description of the coding tree? | P9  Themes and sub-themes given in Table 2 |
| 1. Derivation of themes | Were themes identified in advance or derived from the data? | P7 data analysis sub-heading of methods |
| 1. Software | What software, if applicable, was used to manage the data? | P7 data analysis sub-heading of methods |
| 1. Participant checking | Did participants provide feedback on the findings? | No |
| Reporting | | |
| 1. Quotations presented | Were participant quotations presented to illustrate the themes / findings? Was each quotation identified? e.g. participant number | P9-22 Results |
| 1. Data and findings consistent | Was there consistency between the data presented and the findings? | P9-22 Results |
| 1. Clarity of major themes | Were major themes clearly presented in the findings? | P9-22 Results |
| 1. Clarity of minor themes | Is there a description of diverse cases or discussion of minor themes? | P9-22 Results |
