# Supplementary material for: The Views and Experiences of Integrated Care System Commissioners About the Adoption and Implementation of Virtual Wards in England: Qualitative Exploration Study
Source: J Med Internet Res. 2024 Nov 27;26:e56494. doi: 10.2196/56494 (PMC11635316; doi:10.2196/56494)
Supplement: Multimedia Appendix 2 [file jmir_v26i1e56494_app2.docx]

**Title of Study:** Adoption and implementation of virtual wards in Integrated Care Systems in England: A qualitative exploration of the views and experiences of Integrated Care System commissioners

**Interview Topic Guide**

*(In line with usual practice in qualitative interviewing, the questions asked are likely to evolve as interviews are conducted and key issues emerge)*

Opening statement: *Thank you again for agreeing to talk to me today. I am a researcher from Newcastle University working on an NIHR funded project exploring factors influencing the adoption of virtual wards across integrated care systems in England.* *Throughout the interview when I use the term ‘virtual wards’ I’m referring to virtual wards enabled by technology, i.e. the management of patients via a digital platform (e.g. using remote monitoring technology to monitor patients’ symptoms and physiological parameters). You are free to stop the interview and withdraw your participation at any point without giving a reason.*

*Does that all sound ok to you? Do you have any questions before we start? Ok great, let’s get started…*

1. Please can you briefly describe your job role?
   - *Probes: key tasks/ areas of specialism, employed by whom, years in this job as a commissioner*
2. Please can you tell me about your involvement with virtual wards to date?
   - *Probes: identifying funding, workforce planning (e.g. clinicians to support VWs – new or reassigned? What mix of skills?), Procuring tech/services?*
   - *Involved in implementation plans/ organising virtual wards in practice, discussing virtual wards with patients and other colleagues, delivering care by virtual wards.*
   - *Organisational responsibilities e.g. reporting into NHSE, liaising with other colleagues in other hospitals or community settings across the ICS; quality assurance etc?*
3. Can you please describe the virtual wards you have been involved in organising/ planning/delivering?
   - *Probes: What type of virtual ward e.g. specific condition – frailty, Covid? Main goal of the ward e.g. admission avoidance, early supported discharge, both? Organisation/system leadership (acute led, community led, primary care, system led, integrated model etc). Scope? (monitoring v monitoring and treatment v monitoring, diagnostics & treatment)*
   - *What technology/modality have you used as part of the virtual wards? (e.g. oximeters*,*24 hour wearables v analogue v health surveillance etc). How confident do people delivering feel about the tech? Comparison of tech-enabled VW to non-tech?*
   - *Delivery: Frequency of patient contact (and how contacted – face to face, remote), hours of service (12/24 hour etc), urban vs rural delivery, staff involved etc?*
   - Dedicated VW team or integrated into other teams? Which would be preferable?
   - *Does virtual ward meet national definition/principles i.e. implementing as desired? If (deliberately) not, why?*
4. Can you tell me a little bit about how the virtual wards programme was initially developed?
   - *Probes: Who developed the programme? How did your organisation become involved in implementing virtual wards? How was the decision made to participate/what triggered decision/who was involved in decision-making? Evolved from Covid virtual ward?*
5. Who will lead implementation (or who is leading)?
   - *What attributes/qualities does this person have/lack to do this role effectively? (if enthusiasm comes up here, probe what drives this – personality, circumstantial factors etc)*
   - *Influence of other organisations in making decision to implement virtual wards?*
6. Can you describe the plan for implementing virtual wards?
   - *Probes: How detailed? Who knows about it? Do you feel it is realistic/feasible? Contingency plans? Timeframes/anticipated milestones? What support is available? Sustainability of model? Workforce – new hires/reassigned from other duties, how handle both? If reassignment – how does this work, how do people feel about it?*
7. What do you see as the advantages/disadvantages of virtual wards compared to current practice? (e.g. for patients/carers/clinical practice/practitioners/trusts)
   - *Probes: Advantages/disadvantages for patients? Challenges of patients using virtual wards (e.g. digital literacy issues)? Perceived implications for health inequalities? e.g. what if home is a risk? Perceived impact on carers? Impact on clinical outcomes?*
   - *Barriers and challenges to delivering/implementing virtual wards from HCP/trust perspectives? Personal misgivings e.g. fear of reduced credibility of care providers? How may affect professional identity?*
8. In your view, what would make virtual wards successful?
   - *Probes: Characteristics of individuals involved in implementing? (if enthusiasm comes up here, probe what drives this – personality, circumstantial factors etc?) Communication factors? Skills/resources (this is likely to be what focused on, try to elicit other factors). How can any barriers be overcome? Is there anything NHS can do differently to help e.g. any cultural changes?*
   - *Successful for who/what -* patients or staff, systems, patient flow, finance?
9. Why do you think some areas are further ahead than others in implementing virtual wards?
   - *Is this solely about money? Or something else? (e.g. clinical leadership, trust, risk…)*
10. What are your personal feelings around the adoption/implementation of virtual wards?
    - *Probes: Any emotions around this e.g. sense of achievement, important career milestone, burdensome, worried, optimistic, excited, fear around sanctions if not adopted? What worries you?*
    - *How confident are you in the evidence for VWs? What evidence is this/where from?*
    - *Where do you see the future of virtual wards? Potential for long-term cost-saving?*

*Closing questions*

1. Is there anything else you would like to tell us about virtual wards? Anything that we’ve not already asked you about? Or were any of our questions less relevant/important?
2. Can you suggest any other individuals who would be good to speak to for this research?
3. Are you happy for a follow-up (interview/focus group)? What would be the best interval e.g. when might meaningful milestones be reached?
