# Supplementary material for: The Views and Experiences of Integrated Care System Commissioners About the Adoption and Implementation of Virtual Wards in England: Qualitative Exploration Study
Source: J Med Internet Res. 2024 Nov 27;26:e56494. doi: 10.2196/56494 (PMC11635316; doi:10.2196/56494)
Supplement: Multimedia Appendix 3 [file jmir_v26i1e56494_app3.docx]

**Description of Virtual Wards across ICSs**

Though there was a varied level of familiarity with the precise VW models and implementation plans due to differing roles, all participants provided insights about the on-going planning and development work taking place within and across systems. Participants described VWs as a mix of both step-up and step-down services. ‘Step-up’ services were described in relation to ‘admission avoidance’, or ‘alternative admissions’ and involved unwell patients being admitted onto a VW rather than a hospital bed, with referrals typically made via a GP, A&E or self-referral. ‘Step-down’ services (referred to as supporting early discharge) involved providing previously hospitalised patients with treatment (such as IV antibiotics) or close monitoring to aid recovery in their own homes rather than prolonging their hospital stay, with referrals typically made through secondary care consultants.

Most ICS plans focused on VWs for specific health conditions, namely respiratory and frailty conditions, as per the NHSE instruction. Most reported that their respiratory VWs were comparatively more established than frailty, with one ICS reporting they had at least 6 cohorts of patients through the respiratory VW, and another had run a Hospital at Home service for respiratory patients for 7 years. Plans for frailty VWs were generally less advanced.

Whilst the primary focus of the VWs being developed were for respiratory and frailty, some ICSs intended to expand their existing remote monitoring services. This included end of life services, to reduce the numbers of people dying in hospital or being admitted within the last three months of life. Other health services considered to fall within the remit of the VWs programme included patients undergoing chemotherapy, radiotherapy and immunotherapy. One system planned to develop a multi-speciality (or a ‘condition agnostic’) model, for patients aged 18 years and above who may have a range of conditions, from heart failure to COPD.
